# Minimum dietary diversity and consumption of ultra-processed foods among Brazilian children 6-23 months of age

**DOI:** 10.1590/0102-311XEN081422

**Published:** 2023-10-20

**Authors:** Elisa Maria de Aquino Lacerda, Neilane Bertoni, Nadya Helena Alves-Santos, Letícia B. Vertulli Carneiro, Raquel Machado Schincaglia, Cristiano Siqueira Boccolini, Inês Rugani Ribeiro de Castro, Luiz Antonio dos Anjos, Talita Lelis Berti, Gilberto Kac, Brazilian Consortium on Child Nutrition, Dayana Rodrigues Farias

**Affiliations:** 1 Instituto de Nutrição Josué de Castro, Universidade Federal do Rio de Janeiro, Rio de Janeiro, Brasil.; 2 Divisão de Pesquisa Populacional, Instituto Nacional de Câncer José Alencar Gomes da Silva, Rio de Janeiro, Brasil.; 3 Instituto de Estudos em Saúde e Biológicas, Universidade Federal do Sul e Sudeste do Pará, Belém, Brasil.; 4 Instituto de Estudos em Saúde Coletiva, Universidade Federal do Rio de Janeiro, Rio de Janeiro, Brasil.; 5 Instituto de Comunicação e Informação Científica e Tecnológica em Saúde, Fundação Oswaldo Cruz, Rio de Janeiro, Brasil.; 6 Instituto de Nutrição, Universidade do Estado do Rio de Janeiro, Rio de Janeiro, Brasil.; 7 Departamento de Nutrição Social, Universidade Federal Fluminense, Niterói, Brasil.

**Keywords:** Child Nutrition, Complementary Feeding, Ultra-processed Foods, Epidemiological Studies, Nutrição da Criança, Alimentação Complementar, Alimentos Ultraprocessados, Estudos Epidemiológicos, Nutrición del Niño, Alimentación Complementaria, Alimentos Ultraprocesados, Estudios Epidemiológicos

## Abstract

The study aimed to estimate the prevalence of minimum dietary diversity (MDD) and consumption of ultra-processed foods in children 6-23 months of age according to sociodemographic variables. Three indicators of complementary feeding of 4,354 children from the *Brazilian National Survey on Child Nutrition* (ENANI-2019) were built based on a questionnaire about food consumption on the day before the interview: MDD, consumption of ultra-processed foods, and MDD without the consumption of ultra-processed foods. The prevalence and 95%CI were calculated, stratified by macroregion; race/skin color, education and work status of the mother or caregiver; enrollment in the Brazilian Income Transfer Program; household food security; sanitation; and child enrollment in daycare/school. The overall prevalence of MDD was 63.4%, with lower prevalences among children who lived in the North Region (54.8%), whose mothers or caregivers had 0-7 years of education (50.6%), and lived under moderate or severe food insecurity (52.6%). Ultra-processed foods were consumed by 80.5% of the children, with the highest prevalence in the North Region (84.5%). The prevalence of MDD without ultra-processed foods was 8.4% and less prevalent among children with black mothers or caregivers (3.6%) and among those whose mother or caregiver had 8-10 years of education (3.6%). The most frequently consumed food groups from the MDD indicator were grains, roots and tubers (90.2%), dairy products (81%) and those from ultra-processed food were sweet or salty cookies/crackers (51.3%) and instant flours (41.4%). The ubiquitous presence of ultra-processed foods in the diets of Brazilian children and the low frequency of diversified foods, especially among the most vulnerable populations, indicate the need to strengthen policies and programs to ensure adequate and healthy infant nutrition.

## Resumo

O objetivo do estudo foi estimar a prevalência de diversidade alimentar mínima (DAM) e consumo de alimentos ultraprocessados em crianças de 6-23 meses de acordo com variáveis sociodemográficas. Três indicadores de alimentação complementar de 4.354 crianças do *Estudo Nacional de Alimentação e Nutrição Infantil* (ENANI-2019) foram construídos com base em um questionário sobre o consumo alimentar do dia anterior à entrevista: DAM, consumo de alimentos ultraprocessados e DAM sem consumo de alimentos ultraprocessados. Foram calculadas as prevalências e IC95%, estratificados por macrorregião; raça/cor da pele, escolaridade e situação profissional da mãe ou cuidador; inscrição no Programa Bolsa Família; segurança alimentar do domicílio; saneamento; e matrícula da criança em creche/escola. A prevalência geral de DAM foi de 63,4%, com menores prevalências entre crianças que residiam na Região Norte (54,8%), cujas maẽs ou cuidadores tinham de 0-7 anos de estudo (50,6%) e entre aquelas que viviam em situação de insegurança alimentar moderada ou grave (52,6%). Os alimentos ultraprocessados foram consumidos por 80,5% das crianças, com maior prevalência na Região Norte (84,5%). A prevalência de DAM sem alimentos ultraprocessados foi de 8,4%, sendo menos prevalente entre crianças cuja mãe ou cuidador era negro (3,6%) e entre aquelas cuja mãe ou cuidador tinha 8-10 anos de estudo (3,6%). Os grupos de alimentos do indicador DAM mais consumidos foram os cereais, raizes e tubérculos (90,2%), os derivados do leite (81%) e os dos alimentos ultraprocessados foram os biscoitos (51,3%) e os cereais instantâneos (41,4%). A onipresença de alimentos ultraprocessados na alimentação das crianças brasileiras e a baixa frequência de diversidade alimentar, especialmente entre as populações mais vulneráveis, indicam a necessidade de fortalecer políticas e programas para garantir uma nutrição infantil adequada e saudável.

## Resumen

El objetivo del estudio fue estimar la prevalencia de diversidad alimentaria mínima (DDM) y consumo de alimentos ultraprocesados en niños de 6-23 meses según variables sociodemográficas. Se construyeron tres indicadores de alimentación complementaria de 4.354 niños de el *Estudio Nacional de Alimentación y Nutrición Infantil* (ENANI-2019) a partir de un cuestionario sobre el consumo de alimentos el día anterior a la entrevista: DDM, consumo de alimentos ultraprocesados y DDM sin consumo de alimentos ultraprocesados. Se calcularon la prevalencia y los IC95%, estratificados por macrorregión; raza/color de piel, situación educativa y laboral de la madre o cuidador; inscripción al Programa Bolsa Familia; seguridad alimentaria del hogar; saneamiento; e inscripción de niños en guarderías/escuelas. La prevalencia general de DDM fue del 63,4%, con prevalencias menores entre los niños que vivían en la Región Norte (54,8%), cuyas madres o cuidadores tenían entre 0-7 años de escolaridad (50.6%) y los que vivían en inseguridad alimentaria moderada o grave (52,6%). Los alimentos ultraprocesados fueron consumidos por el 80,5% de los niños, con mayor prevalencia en la Región Norte (84,5%). La prevalencia de DDM sin alimentos ultraprocesados fue del 8,4%, siendo menos prevalente entre niños de padres negros (3,6%) y con 8-10 años de escolaridad (3,6%). Los grupos de alimentos más consumidos del indicador DDM fueron los granos, raíces y tubérculos (90,2%), y los productos lácteos (81%) y los de alimentos ultraprocesados fueron las galletas (51,3%) y los cereales instantáneos (41,4%). La presencia ubicua de alimentos ultraprocesados en las dietas de los niños brasileños y la baja frecuencia diversidad dietética, especialmente entre las poblaciones más vulnerables, indican la necesidad de fortalecer políticas y programas para garantizar una nutrición infantil adecuada y saludable.

## Introduction

Adequate and safe infant and young child feeding contributes to achieving the potential for growth and development in childhood, forming healthy eating habits, preventing micronutrient deficiencies, obesity, and cardiovascular diseases in adulthood, and reducing infant mortality ^1^ ^,^ ^2^. Dietary diversity refers to the variety of foods eaten and the consequent supply of macro- and micronutrients necessary for the body’s physiological balance ^3^. It is associated with a reduced risk of the child having developmental delays ^4^, short stature ^5^ ^,^ ^6^, thinness ^6^, and developing food allergies ^7^.

A lack of dietary diversity is one of the main problems in children < 2 years old in developing countries, mainly comprising a limited intake of fruits and vegetables ^8^. The minimum dietary diversity (MDD) belongs to the World Health Organization (WHO) set of indicators to monitor infant and young child feeding. It is defined as the proportion of children 6-23 months of age who consumed foods and beverages from at least five out of eight defined food groups during the previous day ^9^. The prevalence of MDD worldwide is very heterogeneous. In 80 low- and middle-income countries, only 21.3% were reported to have an MDD prevalence higher than 50% ^10^. In an analysis of trends from 2010 to 2020 of 50 countries that had MDD data available, only 21 had made improvements in diversifying children’s diets ^8^. Socioeconomic factors, such as access to water ^11^ and basic sanitation ^12^, higher family income, maternal work ^13^ ^,^ ^14^, better wealth status ^14^ ^,^ ^15^ and women’s empowerment ^16^, have been found to be positively associated with MDD in children < 2 years old. Dietary diversity can also be impeded by the increasing consumption of ultra-processed foods, which can replace more nutritious foods in a child’s diet ^17^ ^,^ ^18^.

In adults, ultra-processed foods intake increases the chance of obesity, abdominal obesity, metabolic syndrome, cerebrovascular diseases, gastrointestinal diseases, dyspepsia, cancer, depression, and all-cause mortality ^19^ ^,^ ^20^ ^,^ ^21^. In children and adolescents, ultra-processed foods consumption was found to be related to a worse lipid profile and increased total and LDL-cholesterol and triglycerides ^22^. In Rio de Janeiro, Brazil, the nutritional analysis of ultra-processed foods consumed by children 6-59 months of age showed an unbalanced dietary profile of these foods, with two-thirds having an excess of at least one critical nutrient ^23^.

Among the factors associated with ultra-processed foods consumption by Brazilian children < 10 years old, the following stand out: high ^24^ and low levels of schooling of the children’s mothers ^25^; older age groups ^24^ ^,^ ^25^; nonattendance at daycare/school ^26^; mother’s marital status (single compared to married) ^27^; higher number of residents in the household ^27^; and enrollment in government benefits ^27^.

In Brazil, national data on infant feeding practices are scarce ^28^ ^,^ ^29^. Therefore, this study describes the prevalence of MDD and ultra-processed foods consumption indicators, their components, and the prevalence of MDD without ultra-processed foods consumption among children 6-23 months of age; it also examines each of these food practices according to sociodemographic variables.

## Materials and methods

### Study design, population, and sampling

The *Brazilian National Survey on Child Nutrition* (ENANI-2019) evaluated a sample of 14,558 children < 5 years old from 12,524 households ^30^. It was a household survey with a complex sample, geographic stratification, census sector clustering, and weight calibration. These parameters allow the generation of estimates for Brazil, as well as by macroregion, sex, and age group for children < 5 years old and their households ^31^. For the present study, children 6-23 months of age (≥ 183 and ≤ 730 days of age) were selected (n = 4,354 children).

### Data collection and variables

The questionnaire included items regarding markers of the child’s diet on the day before the interview: breast milk; grains, roots and tubers; legumes; dairy products; flesh foods; eggs; vitamin A-rich fruits and vegetables; fruits and vegetables; sweet or salty cookies/crackers; instant flours; yogurts; carbonated drinks; other sugar-sweetened beverages; candies; processed meats; packaged snacks; processed breads; and instant noodles ^32^.

The sociodemographic variables used were the Brazilian macroregions (North, Northeast, Southeast, South, and Central-West), the educational level of the mother or caregiver of the child (0-7; 8-10; 11; ≥ 12 years of education); the mother or caregiver’s work situation (regular work or a fixed schedule; irregular work or with no set schedule; unemployed and looking for a job, and outside the job market); enrollment in the Brazilian Income Transfer Program (yes; no); food insecurity assessed by the *Brazilian Food Insecurity Scale* (EBIA) considering households with residents < 18 years old (food security; mild insecurity; moderate/severe insecurity) ^33^ ^,^ ^34^; basic sanitation (presence of running water and sewage; presence of running water or sewage; no water nor sewage); child’s enrollment in daycare/school (yes; no); and race/skin color of the mother or caregiver (white; mixed-race; black). Given the low representation of mothers or caregivers with an indigenous background or a yellow race/skin color (Asian descendants) (< 1%), the indicator estimates for these subgroups were not presented. Results regarding dietary diversity and consumption of ultra-processed foods stratified according to macroregion and mother or caregiver race/skin color can also be found elsewhere ^35^. More details related to the questionnaire are available on Alves-Santos et al. ^30^ and on the ENANI-2019 website (https://enani.nutricao.ufrj.br/index.php/materiais/).

### Child feeding indicators

The MDD indicator was calculated based on WHO ^9^: the number of children 6-23 months of age who received at least five out of eight food groups on the day before the interview divided by the total number of children in the same age group. The food groups considered were: (1) breast milk; (2) grains, roots and tubers (bread, rice, pasta, baby cereal, potatoes, other starchy vegetables); (3) beans, nuts and seeds (beans, lentils, peas, chickpeas); (4) dairy products (animal milk, infant formula, yogurt, porridge); (5) flesh foods (animal meat, liver, kidney, heart, sausages, processed meats); (6) eggs; (7) vitamin A-rich fruits and vegetables (carrots, pumpkin, sweet potato, cabbage, spinach, other local dark greens leafy vegetables); and (8) fruits and vegetables. The groups names are defined by the WHO, but the foods actually included are those that appear in parentheses.

The ultra-processed foods consumption indicator was calculated as the number of children 6-23 months of age who received at least one ultra-processed food on the day before the interview divided by the total number of children in the same age group. The ultra-processed foods were grouped into ten categories: (1) sweet or salty cookies/crackers; (2) instant flours (rice, corn, wheat or oatmeal); (3) carbonated drinks; (4) other sugar-sweetened beverages (excluding carbonated drinks) (boxed juice, boxed coconut water, guarana syrup, currant juice, powdered juice, or natural fruit juice with added sugar); (5) candies (confectionery); (6) processed meats (hamburger, ham, mortadella, salami, nugget, sausages or frankfurter); (7) packaged snacks; (8) processed breads (such as flatbread, breadsticks and hamburger buns); (9) instant noodles; and (10) yogurts. Foods from categories 2, 3, 4, 6, 8, and 10 are not on the list of selected unhealthy sentinel foods of the WHO indicator consumption of unhealthy foods for children 6-23 months of age ^9^. 

The indicator MDD without ultra-processed foodsts was calculated considering the combination of the occurrence of MDD with the nonoccurrence of ultra-processed foods consumption, thus characterizing a healthy food marker that was better than MDD alone.

### Data analysis

The prevalence and 95% confidence intervals (95%CI) were calculated. The prevalence between groups presented a statistically significant difference when no overlap of the 95%CI occurred. We calculated the estimates of the absolute number of children considering population totals and the coefficient of variation (CV) of the estimates. We assumed that results with a CV ≤ 30% had an adequate level of precision; otherwise, we interpreted the information with caution. The analyses were performed with the R software (http://www.r-project.org) using the functions of the *srvyr* and *survey* packages, considering the structure of the sampling plan, the weights, and the calibration, to compensate for nonresponses and to match the population estimates with the total known population.

### Ethical considerations

The ENANI-2019 was approved by the Research Ethics Committee of the Clementino Fraga Filho University Hospital of the Federal University of Rio de Janeiro (CAAE n. 89798718.7.0000.5257). Data were collected after a parent or caregiver of the child authorized participation in the study through informed consent form.

## Results

Higher frequencies of children 6-23 months of age were observed in the Southeast (39%) and Northeast (28.2%). Most of the study population had mother or caregivers who declared their skin color or race to be mixed-race (54.5%). More than half (55.7%) of the children had a mother or caregiver with at least 11 years of education, 26.5% had an unemployed mother or caregiver, and 36.8% lived in households where at least one of the residents was enrolled in the Brazilian Income Transfer Program. Almost half of the children (46.5%) lived in households with food insecurity. Approximately 71% of the children lived in households supplied with running water and sewage networks, but 6.9% lived in households without access to these infrastructure services. The percentage of children enrolled in daycare centers or schools was 13.9% (Table 1).

**Table 1:** Frequency of children aged 6-23 months of age in Brazil according to sociodemographic variables, 2019.

Variables	%	95%CI	Children (x 1,000) *
Brazilian macroregions			
North	11.0	10.9; 11.0	486.9
Northeast	28.2	28.1; 28.3	1,250.3
Southeast	39.0	38.8; 39.3	1,729.3
South	13.4	13.4; 13.5	594.5
Central-West	8.3	8.3; 8.4	368.6
Race/Skin color of the mother or caregiver			
White	31.5	28.6; 34.4	1,395.8
Mixed race	54.5	51.5; 57.5	2,415.7
Black	12.7	10.4; 14.9	562.1
Yellow	1.0 **	0.4; 1.6	44.0
Indigenous	0.3 **	0.0; 0.5	12.0
Educational level of the mother or caregiver (years of education)			
0-7	21.3	18.6; 24.0	942.5
8-10	23.0	20.0; 25.9	1,017.9
11	37.7	34.7; 40.7	1.670.3
≥ 12	18.0	15.3; 20.8	798.9
Work situation of the mother or caregiver			
Regular work or fixed schedule	21.9	19.0; 24.7	968.5
Irregular work or with no set schedule	14.0	11.7; 16.3	620.1
Unemployed and looking for a job	26.5	23.3; 29.6	1,173.3
Outside the job market	37.7	34.2; 41.1	1,667.8
Brazilian Income Transfer Program			
Yes	36.8	32.7; 40.9	1,630.0
No	63.2	59.1; 67.3	2,799.6
*Brazilian Food Insecurity Scale* (EBIA)			
Food security	53.5	47.9; 59.0	2,368.0
Mild insecurity	36.6	31.9; 41.3	1,622.3
Moderate/Severe insecurity	9.9	8.2; 11.6	439.3
Basic santitation			
Running water and sewage	71.6	67.4; 75.8	3,170.0
Running water or sewage	21.5	18.2; 24.8	953.3
Neither water nor sewage	6.9	3.7; 10.2	306.4
Child enrolled in daycare or school			
No	86.1	83.0; 89.1	3,811.8
Yes	13.9	10.9; 17.0	617.8

95%CI: 95% confidence interval.

* Children (x 1,000): indicates that the value presented in each table cell must be multiplied by 1,000 to obtain the total population of children 6-23 months of age in that condition;

** Coefficient of variation (CV) > 30. CV is a measure of dispersion that indicates data heterogeneity, obtained by the ratio between the standard error and the estimated value of the indicator.

**Figure 1:**
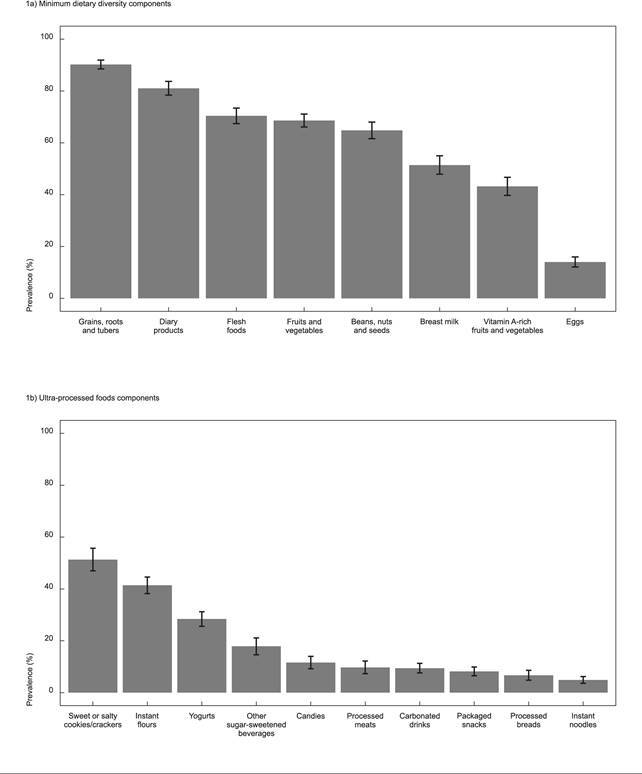
Frequency of consumption of food groups that are components of the indicators minimum dietary diversity (MDD) and ultra-processed foods consumption for children 6-23 months of age in Brazil, 2019.

The most consumed food groups were grains, roots and tubers (90.2%), dairy products (81%), and the least consumed was eggs (14%). The prevalence of breast milk consumption was 51.4% (Figure 1a). The most consumed ultra-processed groups were sweet or salty cookies/crackers (51.3%) and instant flours (41.4%). The prevalence of children’s consumption of any type of sweetened beverage (including carbonated drinks) was 24.5% (95%CI: 21.1; 27.8) (data not presented). Sweetened beverages (except soda) were consumed by 17.9% of children, and candies (confectionery) were consumed by 11.6% of them. The prevalence of consumption was less than 10% for each of the following groups: processed meats, carbonated drinks, packaged snacks, processed breads, and instant noodles (Figure 1b).

The prevalence of MDD was 63.4%, with the highest prevalence in the Southeast (69.4%; 95%CI: 62.8; 76.0) and the lowest in the North Region (54.8%; 95%CI: 47.4; 62.1), with statistically significant differences (Table 2). There was a gradient in the prevalence of MDD based on the educational level of the person responsible for the child and statistically significant differences were found among children whose mothers or caregivers had a lower educational level (≤ 7 years of education), the prevalence was 50.6% (95%CI: 45.0; 56.1), and those whose mothers or caregivers had ≥ 12 years of education, the estimate was 76.5% (95%CI: 68.9; 84.1). The prevalence of MDD among children whose mothers or caregivers worked regular or fixed hours was higher than that of the other categories, but statistically significant differences were observed only between regular or fixed-hour work (71.8%; 95%CI: 65.9; 77.6) and out of the job market (61.2%; 95%CI: 56.6; 65.7). Children not enrolled in the Brazilian Income Transfer Program had higher MDD (67.3%; 95%CI: 63.1; 71.5) than those enrolled (56.7%; 95%CI: 51.4; 61.9). There was no statistically significant difference in the prevalence of MDD by race/skin color of the mother or caregivers, EBIA categories, basic sanitation conditions, and enrollment in daycare/school (Table 2).

**Table 2:** Frequency of minimum dietary diversity (MDD) among children 6-23 months of age in Brazil and according to sociodemographic variables, 2019.

Variables	%	95% CI	Children (x 1,000) *
Brazil	63.4	59.9; 66.9	2,807.4
Brazilian macroregions			
North	54.8	47.4; 62.1	266.8
Northeast	57.3	50.5; 64.0	716.0
Southeast	69.4	62.8; 76.0	1,199.7
South	64.4	56.5; 72.2	382.7
Central-West	65.7	60.4; 71.1	242.2
Race/Skin color of the mother or caregiver			
White	66.5	61.2; 71.8	928.1
Mixed race	63.0	58.6; 67.3	1,521.5
Black	60.6	52.8; 68.3	340.4
Educational level of the mother or caregiver (years of education)			
0-7	50.6	45.0; 56.1	476.5
8-10	58.3	52.2; 64.3	593.2
11	67.4	63.7; 71.2	1,126.3
≥ 12	76.5	68.9; 84.1	611.4
Work situation of the mother or caregiver			
Regular work or fixed schedule	71.8	65.9; 77.6	694.9
Irregular work or with no set schedule	61.4	53.5; 69.2	380.6
Unemployed and looking for a job	60.7	55.4; 65.9	711.9
Outside the job market	61.2	56.6; 65.7	1,019.9
Brazilian Income Transfer Program			
Yes	56.7	51.4; 61.9	923.6
No	67.3	63.1; 71.5	1,883.8
*Brazilian Food Insecurity Scale* (EBIA)			
Food security	65.1	60.1; 70.0	1,540.4
Mild insecurity	63.9	58.3; 69.4	1,035.9
Moderate/Severe insecurity	52.6	43.7; 61.5	231.1
Basic sanitation			
Running water and sewage	65.0	60.5; 69.5	2,061.3
Running water or sewage	59.3	53.7; 64.9	565.4
Neither running water nor sewage	59.0	44.0; 74.0	180.7
Child enrolled in daycare or school			
No	63.1	59.7; 66.4	2,403.7
Yes	65.3	55.2; 75.4	403.7

95%CI: 95% confidence interval.

Note: MDD - number of children 6-23 months of age who received at least five out of eight food groups on the day before the interview, divided by the total number of children in the same age group [food groups: (1) breast milk; (2) grains, roots and tubers; (3) beans, nuts and seeds; 4) dairy products; (5) flesh foods; (6) eggs; (7) vitamin A-rich fruits and vegetables; and (8) fruits and vegetables].

* Children (x 1,000): indicates that the value presented in each table cell must be multiplied by 1,000 to obtain the total population of children 6-23 months of age in that condition.

The prevalence of consumption of ultra-processed foods was 80.5%, with the highest prevalence in the North Region (84.5%; 95%CI: 82.0; 87.0) and the lowest in the South (76.8%; 95%CI: 72.3; 81.4) and Central-West (76.1%; 95%CI: 70.6; 81.6) regions. Almost 85% of children whose mothers or caregivers had between 8-10 years of education and 73.4% of children whose parents had higher educational levels (≥ 12 years of education) consumed ultra-processed foods. The consumption of ultra-processed foods was high (> 77%) in all socioeconomic strata, and there were no statistically significant differences when comparing the prevalence of different categories of race/skin color, food security, and enrollment in the Brazilian Income Transfer Program, or basic sanitation conditions (Table 3).

**Table 3:** Frequency of ultra-processed foods intake for children 6-23 months of age in Brazil and according to sociodemographic variables, 2019.

Variables	%	95%CI	Children (x 1,000) *
Brazil	80.5	77.2; 83.8	3,566.0
Brazilian macroregions			
North	84.5	82.0; 87.0	411.4
Northeast	82.0	76.7; 87.2	1,024.9
Southeast	80.5	73.2; 87.8	1,392.4
South	76.8	72.3; 81.4	456.8
Central-West	76.1	70.6; 81.6	280.5
Race/Skin color of the mother or caregiver			
White	78.1	72.8; 83.5	1,090.4
Mixed race	81.0	77.6; 84.3	1,955.8
Black	83.7	78.0; 89.4	470.7
Educational level of the mother or caregiver (years of education)			
0-7	81.2	76.1; 86.4	765.5
8-10	84.8	81.2; 88.5	863.7
11	80.8	75.8; 85.9	1,350.3
≥ 12	73.4	67.8; 79.0	586.6
Work situation of the mother or caregiver			
Regular work or fixed schedule	78.7	71.4; 86.0	762.3
Irregular work or with no set schedule	87.9	83.0; 92.7	545.0
Unemployed and looking for a job	82.1	78.1; 86.1	963.2
Outsite the job market	77.7	73.1; 82.3	1,295.5
Brazilian Income Transfer Program			
Yes	81.7	78.3; 85.1	1,331.3
No	79.8	75.7; 84.0	2,234.7
*Brazilian Food Insecurity Scale* (EBIA)			
Food security	79.6	75.4; 83.8	1,884.8
Mild insecurity	81.9	78.2; 85.5	1,328.2
Moderate/Severe insecurity	80.4	70.7; 90.0	353.0
Basic sanitation			
Running water and sewage	79.8	75.3; 84.4	2,530.2
Running water or sewage	82.4	78.3; 86.5	785.3
Neither running water nor sewage	81.8	71.7; 91.8	250.5
Child enrolled in daycare or school			
No	79.5	75.6; 83.4	3,030.6
Yes	86.7	80.3; 93.0	535.4

95%CI: 95% confidence interval.

Note: ultra-processed foods - number of children 6-23 months of age who received at least one ultra-processed food on the day before the interview, divided by the total number of children in the same age group [food groups: (1) sweet or salty cookies/crackers; (2) instant flours; (3) carbonated drinks; (4) other sugar-sweetened beverages; (5) candies; (6) processed meats; (7) packaged snacks; (8) processed breads; (9) instant noodles; and (10) yogurts];

* Children (x 1,000): indicates that the value presented in each table cell must be multiplied by 1,000 to obtain the total population of children 6-23 months of age in that condition.

The prevalence of adequate feeding (MDD without ultra-processed foods) was 8.4% among children 6-23 months of age. The North Region showed the lowest prevalence (3.7%; 95%CI: 1.9; 5.5), and the Central-West (12.1%; 95%CI: 8.9; 15.3) and the South (10.4%; 95%CI: 7.4; 13.3) regions showed the highest prevalence. Statistically significant differences were found between North and Southeast, South, and Central-West, and between Northeast and Central-West. The prevalence of this indicator among children with white mothers or caregivers was approximately 3 times higher than that among children with black ones (11.2%; 95%CI: 7.3; 15.1 vs. 3.6%; 95%CI: 1.7; 5.6). Children whose mothers or caregivers had irregular work had the lowest prevalence of adequate feeding compared to those out of the job market (3.8%; 95%CI: 1.6; 6.1 vs. 9.9%; 95%CI: 6.6; 13.3). Despite adequate feeding being lower in households without running water and sewage than in households with both services, the CV > 30 indicates data heterogeneity. There were no statistically significant differences when comparing the prevalence of adequate feeding according to enrollment in daycare/school (Table 4).

**Table 4:** Frequency of minimum dietary diversity (MDD) without consumption of ultra-processed foods for children 6-23 months of age in Brazil and according to sociodemographic characteristics, 2019.

Variables	%	95%CI	Children (x 1,000) *
Brazil	8.4	6.6; 10.1	371.0
Brazilian macroregions			
North	3.7	1.9; 5.5	18.0
Northeast	6.0	3.4; 8.7	75.5
Southeast	9.9	6.0; 13.8	171.4
South	10.4	7.4; 13.3	61.6
Central-West	12.1	8.9; 15.3	44.6
Race/Skin color of the mother or caregiver			
White	11.2	7.3; 15.1	156.5
Mixed race	8.0	6.2; 9.9	193.3
Black	3.6	1.7; 5.6	20.4
Educational level of the mother or caregiver (years of education)			
0-7	5.3	2.8; 7.8	49.9
8-10	3.6	1.8; 5.4	36.9
11	9.5	5.7; 13.3	158.7
≥ 12	15.7	11.2; 20.2	125.5
Work situation of the mother or caregiver			
Regular work or fixed schedule	9.8	5.9; 13.7	95.3
Irregular work or with no set schedule	3.8	1.6; 6.1	23.8
Unemployed and looking for a job	7.3	4.8; 9.9	86.1
Outside the job market	9.9	6.6; 13.3	165.9
Brazilian Income Transfer Program			
Yes	6.2	3.5; 9.0	101.8
No	9.6	7.7; 11.6	269.2
*Brazilian Food Insecurity Scale* (EBIA)			
Food security	9.2	6.9; 11.4	216.7
Mild insecurity	7.2	4.8; 9.6	116.9
Moderate/Severe insecurity	8.5 **	0.7; 16.3	37.4
Basic sanitation			
Running water and sewage	9.6	7.3; 11.8	303.6
Running water or sewage	6.4	3.6; 9.1	60.9
Neither running water nor sewage	2.1 **	0.0; 4.8	6.4
Child enrolled in daycare or school			
No	9.0	6.9; 11.0	341.5
Yes	4.8 **	1.6; 7.9	29.4

95%CI: 95% confidence interval.

* Children (x 1,000): indicates that the value presented in each table cell must be multiplied by 1,000 to obtain the total population of children 6-23 months of age in that condition;

** Coefficient of variation (CV) > 30. CV is a measure of dispersion that indicates data heterogeneity, obtained by the ratio between the standard error and the estimated value of the indicator.

## Discussion

More than 80% of Brazilian children < 2 years old consumed ultra-processed foods, almost two-thirds consumed a minimally diversified diet, and less than 10% of children ate a diverse diet without ultra-processed foods. The MDD prevalence was lower among children living in the North compared with the Northeast Region, among children whose mothers or caregivers had lower educational levels (8-10 compared with ≥ 12 years of education), and were out of the job market compared with those with regular or fixed-hour work. 

Considering 11 countries from Latin America and the Caribbean, the prevalence of MDD varied from 25.4% to 72.9%. Brazil is situated in an intermediate position, with the prevalence of MDD (63.4%) being higher than that of Haiti (25.4%), Guyana (40.3%), Paraguay (52.1%), Dominican Republic (51.3%), Belize (57.8%), Honduras (60.7%), and Guatemala (62.8%), but lower than that of Mexico (69.4%), Bolivia (70.5%), and El Salvador (72.9%) ^36^.

ENANI-2019 shows that children whose mothers or caregivers had higher educational levels (≥ 12 years of education) had a prevalence of MDD that was approximately 50% higher (76.5% vs. 50.6%) than that of children whose mothers or caregivers had lower educational levels (0-7 years). Differences in MDD prevalence according to education were also found in other studies in Brazil ^37^ and lower- and middle-income countries ^38^ ^,^ ^39^ ^,^ ^40^ ^,^ ^41^. Mothers or caregivers with higher educational levels are likely to have more knowledge about child nutrition ^39^ ^,^ ^40^ ^,^ ^41^ ^,^ ^42^, more access to media and health information ^13^ ^,^ ^40^ ^,^ ^43^, and they can better understand the messages conveyed about child nutrition ^13^. 

The observed high prevalence of meeting MDD among children whose mothers had regular work is consistent with previous studies ^44^ ^,^ ^45^. Studies on MDD and the Brazilian Income Transfer Program program were not identified, but worse eating habits were described in school children whose families receive the Brazilian Income Transfer Program ^46^.

Among the foods that compose the MDD indicator, eggs, a low-cost option that contains protein of high biological value and reasonable amounts of iron, and vitamin A, were consumed by only 14% of Brazilian children 6-23 months of age. The prevalence of egg consumption in the previous 24 hours for children < 24 months, based on nationally representative surveys conducted from 2004-2011, was 11.9% in Africa, 28.9% in Asia, and 37.2% in Latin America and the Caribbean ^47^. A possible explanation, which may partially explain our findings, are the lingering effects of the traditional recommendation, currently in disuse, of avoiding its consumption before one year old to prevent food allergies ^47^ ^,^ ^48^.

The high consumption of ultra-processed foods found by ENANI-2019 beginning in the first year of life reveals early exposure to dietary components such as sugar, sodium, and saturated fat, which are associated with the incidence of noncommunicable chronic diseases ^21^, as well as to food additives. Emulsifiers such as carboxymethylcellulose and polysorbate-80 were found to induce low-grade inflammation even at low concentrations and metabolic syndrome/obesity, related to changes in the intestinal microbiota ^49^. Residues of substances in ultra-processed food packaging, such as phthalates and bisphenol, can pass into the food and act as endocrine disruptors associated with harmful effects ^50^.

A study in Brasília, Brazil, with 847 children in primary health care showed that the prevalences of ultra-processed food consumption were 56.3% and 86.3% among children 6-12 and 12-24 months of age, respectively ^50^. In another study in Brazil (Viçosa, Minas Gerais State) with 231 children 6-24 months of age, the prevalence of ultra-processed and processed foods consumption was 94%. The energy contribution from these foods in the diet was higher among children > 12 months than in children from 6-12 months of age and among those who were not breastfed ^51^. Neither study allows adequate comparison with ENANI-2019. The first study included different age groups and used another definition of the ultra-processed food indicator, and the second included ultra-processed and processed foods in the same group. In addition, both studies considered a self-selected sample, while ENANI-2019 included a nationally representative sample.

Sweet or salty cookies/crackers and instant flours were consumed by 51.3% and 41.4% of children < 2 years old, respectively. Sweet or salty cookies/crackers are offered for breakfast, snacks, and supper; instant flours are also used to prepare porridges and are considered as healthy food by families and health professionals. In general, both products contain sugar in their formulations and, based on the literature ^52^, we hypothesize that their intake may have displaced the consumption of natural foods, such as grains, roots, tubers, and fruits, which may affect the diversity and quality of a child’s diet.

The finding on the high prevalence of children who drank other sugar-sweetened beverages is consistent with a study carried out with national data from 51 countries, which showed that the consumption of these beverages among children over two years of age and adolescents ranged from 115mL/day in Australia to 710mL/day in China. This intake is considered excessive and indicates the need for policy control ^53^. Some meta-analyses revealed that the consumption of these beverages is associated with a greater chance of weight gain ^54^, symptoms of attention-deficit hyperactivity disorder ^55^, increased systolic blood pressure and hypertension ^56^, dyslipidemia ^57^, and overweight and obesity when consumption of sweetened beverages is higher than four times a week ^58^. The prevalence of other sugar-sweetened beverage intake was more elevated than that of carbonated drinks. Considering that most sugar-sweetened beverages contain fruit juice as an ingredient, families may consider these beverages to be healthier than carbonated drinks, even though both are ultra-processed foods containing excessive amounts of sugar ^59^.

Ultra-processed foods are hyperpalatable and highly convenient due to the technological processes and ingredients used in their manufacture. In nutritional terms, they commonly provide fewer nutrients than unprocessed or minimally processed foods ^60^. Evidence from representative studies of 11 countries between 2001 and 2015 shows that the greater inclusion of ultra-processed foods in the diet is associated with worse diet quality ^60^.

Evidence on ultra-processed food consumption and health consequences in childhood is still scarce. A cross-sectional study in Brasília with 538 children 6-24 months of age treated at primary health units showed that ultra-processed foods and processed foods accounted for one-third of energy intake. The greater their consumption, the greater the intake of saturated fat, sugar, and sodium ^61^. A randomized clinical trial with 308 low-income Brazilian children showed an increase in the energy contribution of ultra-processed foods from 3-6 years old. Those in the highest tertile of ultra-processed foods consumption at three years of age had higher concentrations of total cholesterol and triglycerides at age six than those in the lowest tertile ^62^.

In the present study, the North presented a higher prevalence of ultra-processed food consumption than the South and Central-West regions, and there were no statistically significant differences in the other variables. This finding shows that the consumption of these foods is widespread in different socioeconomic strata in Brazil and may result from corporate political activity and promotional strategies from food industries to increase sales and profits ^63^. This political activity refers to corporations’ attempts to influence public policy and public opinion in their favor. The main strategies are coalition management, information management, direct involvement and influence in policy, and legal action ^64^. Therefore, it can be said that some regulatory measures are in place but are still insufficient to protect children from industry practices.

Regarding the third indicator studied, MDD without ultra-processed food consumption, which was considered here to be a better indicator of dietary quality than diversity alone, a very low prevalence was observed, especially in children of black mothers or caregivers (3.6%), among children whose mothers or caregivers had 8-10 years of education (3.6%) compared to 11 (9.5%) and ≥ 12 years (15.7%), and among children whose mothers or caregivers had irregular work and no fixed hours (3.8%) compared to those out of the job market (9.9%). It is not possible to compare these results with those of other studies because this is the first time that this indicator has been presented. However, the groups with lower prevalence rates were, in general, the most vulnerable, except for educational level, following the same scenario observed for the MDD indicator.

The promotion of healthy eating is one of the priorities of the public policies of the Brazilian government. The *Dietary Guidelines for Brazilian Children Under 2 Years of Age* ^65^ published in 2019 included the issue of dietary diversity as one of the main recommendations for complementary feeding. The guidelines reinforced the recommendation to not offer ultra-processed foods in the first two years of life, considering all the evidence on the effects of ultra-processed food consumption on health and the environment ^60^ ^,^ ^65^. In addition to promoting healthy eating, governments must oppose the commercial interests of transnational food industries and implement strategies to control and reduce the manufacture, sale, and promotion of these foods and discourage their consumption. Civil society organizations and social movements should be allies in this process by pressuring the government to implement control policies ^63^ ^,^ ^66^ ^,^ ^67^ ^,^ ^68^. In Brazil, between 2016 and 2019, 84 bills aimed at regulating food industry practices were under consideration in the Brazilian legislature. Nevertheless, none of the proposed policies were enacted by 2020 ^69^. 

The study’s strengths include the fact that the ENANI-2019 offers unprecedented results on two crucial infant feeding indicators and the proposition of another indicator that reflects the quality of food. Another strength is the sample size, which allowed an accurate estimation of the indicators. One of the study’s limitations is inherent to the type of questionnaire used, as it collects only qualitative dietary data, can not represent habitual intake of an individual person, is not designed to collect information on within-person variation, and relies on respondents’ memory ^70^. However, the instrument is easy to use and recommended for monitoring children’s diets of large representative samples of the population worldwide, allowing comparability ^9^ ^,^ ^70^. Concerning the ultra-processed food consumption indicator, its definition included ten food groups classified as ultra-processed foods by NOVA ^71^, accurately capturing the consumption of this food group. However, for two of these groups - sweet or savory biscuits/cookies and yogurts - it was not possible to differentiate whether they were unprocessed/processed or ultra-processed, which may have impacted the magnitude of the prevalence of this indicator. Nevertheless, we believe that the estimation error was small, considering that homemade biscuits/cookies and yogurts were not frequently reported in the 24-hour food recalls of children from ENANI-2019 (data yet to be published). Also, the ENANI-2019 questionnaire did not have a question about the consumption of chocolate-flavored dairy drinks or other flavors, another ultra-processed food. Therefore, this food was not registered, or mothers reported it within the milk group. We recommend a better standardization of the ultra-processed food consumption indicator used in epidemiological studies and a better definition of foods that compose the WHO’s indicator of consumption of unhealthy foods ^9^.

## Conclusions

Among Brazilian children 6-23 months of age, the prevalence of those who consumed ultra-processed foods and those who had MDD were high, while the prevalence of those who had MDD without ultra-processed foods was very low. For this last indicator, which reflects a more adequate and healthy diet, the prevalence was lower in groups with greater socioeconomic vulnerability.
